# Data set of multi-objective optimization of diesel engine parameters

**DOI:** 10.1016/j.dib.2019.104184

**Published:** 2019-06-24

**Authors:** R. Sathish Kumar, K. Sureshkumar

**Affiliations:** a Department of Automobile Engineering, Hindustan Institute of Technology and Science, Chennai 603103, Tamil Nadu, India; b Department of Mechanical Engineering, SRM Institute of Science and Technology, Kattankulathur, 603203, Tamil Nadu, India

**Keywords:** Multi-objective optimization, Grey relational analysis, Orthogonal array, Design of experiments, Analysis of variance, Diesel engine

## Abstract

This data article presents the experimental data set on the optimization of four important parameters which are type of blending fuel, blending ratio, compression ratio and injection timing for four objective functions namely higher brake thermal efficiency, lower brake specific fuel consumption, lower oxides of nitrogen emission and lower unburnt hydrocarbon emission using grey relational analysis and orthogonal array based experimental design. Each parameter was fixed with three levels and L9 orthogonal array has been chosen for experimental analysis. The data obtained from the experimental work reported that butanol as blending fuel, 40% of maximum blending ratio, compression ratio of 16:1 and injection timing of 26 °CA before top dead centre were identified as optimized set of parameters.

**Table:** Specifications table

Subject area	*Automotive Engineering, Internal combustion Engines*
More specific subject area	*Optimization of diesel engine performance*
Type of data	*Tables and Figures*
How data was acquired	*Experimental analysis in engine testing laboratory*
Data format	*Raw and analyzed data*
Experimental factors	*Multi-objective optimization of diesel engine parameters using grey relational analysis and orthogonal array based design of experiments*
Experimental features	*Set of experiments performed in single cylinder, four stroke direct injection compression ignition engine based on L9 orthogonal array design*
Data source location	*Engine testing laboratory, Department of Automobile Engineering, Hindustan Institute of Technology and Science*
Data accessibility	*Data is with this article*
Related research article	*R. Sathish Kumar, K. Suresh Kumar, R. Velraj. Combustion, Performance and emission characteristics of an unmodified diesel engine fuelled with Manilkara Zapota Methyl Ester and its diesel blends. Applied Thermal Engineering 139 (2018) 196–202.*

**Table:** 

**Value of the data** • This data set provides the optimized values of four key parameters on multi-objective functions for complete replacement of diesel fuel • This data set will be useful for the design of engine parameters for biodiesel applications • This data set will be helpful for the new researchers to understand the effects of four key parameters on four performance objectives

## 1 Data

This data article is presenting the data set on parametric optimization of four key parameters such as compression ratio, type of blending fuel, blending ratio and injection timing for four different objectives namely higher brake thermal efficiency, lower brake specific fuel consumption, lower oxides of nitrogen emission and lower unburnt hydrocarbon emission using grey relational analysis and orthogonal array based experimental design and statistical analysis. Experimental design was carried out using L9 orthogonal array and experimental results were given in Table 6 [1]. Further grey relational analysis was performed and related data were present in Table 7, Table 8. The mean analysis and range analysis were conducted and results were plotted in Fig. 4, Fig. 5 respectively. Finally percentage contribution of each parameter was calculated and presented in Table 10. Confirmation test was conducted results were given in Table 11 [2].

**Table 1:** Selected parameters and levels.

Parameters		Levels		
		1	2	3
A	Type of Blending Fuel	Methanol	Ethanol	Butanol
B	Blending Ratio (%)	20	30	40
C	Compression Ratio	14	16	18
D	Injection Timing (°CA)	20	23	26

**Table 2:** Selected L_9_ orthogonal array for four parameters at three levels (3^4^).

Experiment no.	Parameters and their levels			
	A	B	C	D
1	1	1	1	1
2	1	2	2	2
3	1	3	3	3
4	2	1	2	3
5	2	2	3	1
6	2	3	1	2
7	3	1	3	2
8	3	2	1	3
9	3	3	2	1

**Table 3:** Physicochemical properties of test fuels.

Properties	Diesel	PPME	Methanol	Ethanol	Butanol
Chemical formula	C_10_H_18_	Fatty acid group of C6-C24	CH_3_OH	C_2_H_5_OH	C_4_H_9_OH
Density (gm/cc)	0.83	0.873	0.792	0.789	0.81
Kinematic viscosity (mm^2^/s)	3.11	7.82	0.59	1.52	3.6
Calorific value (MJ/kgK)	41860	38600	22700	26800	33100
Cetane number	48	45	5	8	25
Flash point	63	135	11	14	35

**Table 4:** Specifications of test engine.

Details	Specification
Engine	Single cylinder, 4 stroke, water cooled
Make	Kirloskar
Stroke	110 mm
Bore	87.5 mm
Capacity	661 cc
Power	3.5 kW
Rated speed	1500 rpm
Compression Ratio range	12:1 to18:1
Injection variation	0 - 30º CA BTDC (23 °CA BTDC standard)
Dynamometer	Eddy current, water cooled
Piezo sensor	Range 350 Bar, (Combustion & Diesel injection line)
Crank angle sensor	Resolution 1 Deg, Speed 5500 rpm with TDC pulse.
Data acquisition device	NI USB-6210, 16-bit, 250kS/s.
Temperature sensor	Type K Thermocouple
Load sensor	Strain gauge type Load cell, range 0–50 Kg

**Table 5:** List of instruments, range of measurement, accuracy, measurement technique and percentage uncertainties.

Name of the instrument	Range of measurement	Accuracy	Measurement technique	Percentage uncertainties
AVL 444 – 5 gas analyzer	CO – 0–10% vol	0.01% vol	NDIR principle	±0.2
	CO_2_ – 0 - 20% vol	0.1% vol	NDIR principle	±0.15
	HC – 0 – 20000 ppm	10 ppm vol	Electrochemical	±0.05
	NO_x_ – 0 – 5000 ppm	1 ppm vol	Electrochemical	±0.02
EGT indicator	0–1000 °C	±1 °C	K type thermocouple	±0.15
Engine speed measuring unit	0–10000 rpm	±10 rpm	Magnetic pickup	±0.1
Load measuring unit	0–50 kg	±0.1 kg	Strain gauge type load cell	±0.2
Fuel Measuring burette	0–100 cm^3^	±0.1 cm^3^		±0.1
Stop watch		±0.6 sec		±0.2
Air flow meter	0.2–20 m/s	±0.1 m/s	Hot wire anemometer	±0.1

**Table 6:** Experimental conditions and response sequence.

Experiment Number	Parameters				Response Sequence			
	A	B	C	D	BTE (%)	BSFC (kg/kW-hr)	NO_x_ (ppm)	UBHC (ppm)
1	M	20	14	20	25.76	0.54	897	80
2	M	30	16	23	24.93	0.77	703	93
3	M	40	18	26	26.05	0.52	690	67
4	E	20	16	26	25.85	0.48	711	59
5	E	30	18	20	24.07	0.59	686	90
6	E	40	14	23	20.37	0.75	761	67
7	B	20	18	23	28.28	0.47	803	85
8	B	30	14	26	27.96	0.49	813	89
9	B	40	16	20	29.81	0.44	786	51

**Table 7:** Normalized sequence and deviation sequence.

Experiment Number	Normalized Sequence				Deviation Sequence			
	BTE (%)	BSFC (kg/kW-hr)	NO_x_ (ppm)	UBHC (ppm)	BTE (%)	BSFC (kg/kW-hr)	NO_x_ (ppm)	UBHC (ppm)
1	0.5710	0.6970	0.0000	0.3095	0.4290	0.3030	1.0000	0.6905
2	0.4831	0.0000	0.9194	0.0000	0.5169	1.0000	0.0806	1.0000
3	0.6017	0.7576	0.9810	0.6190	0.3983	0.2424	0.0190	0.3810
4	0.5805	0.8788	0.8815	0.8095	0.4195	0.1212	0.1185	0.1905
5	0.3919	0.5455	1.0000	0.0714	0.6081	0.4545	0.0000	0.9286
6	0.0000	0.0606	0.6445	0.6190	1.0000	0.9394	0.3555	0.3810
7	0.8379	0.9091	0.4455	0.1905	0.1621	0.0909	0.5545	0.8095
8	0.8040	0.8485	0.3981	0.0952	0.1960	0.1515	0.6019	0.9048
9	1.0000	1.0000	0.5261	1.0000	0.0000	0.0000	0.4739	0.0000

**Table 8:** Grey relational coefficient and grey relational grade.

Experiment Number	Grey Relational Coefficient				Grey Relational Grade
	BTE (%)	BSFC (kg/kW-hr)	NO_x_ (ppm)	UBHC (ppm)	
1	0.5382	0.6226	0.3333	0.4200	0.4785
2	0.4917	0.3333	0.8612	0.3333	0.5049
3	0.5566	0.6735	0.9635	0.5676	0.6903
4	0.5438	0.8049	0.8084	0.7241	0.7203
5	0.4512	0.5238	1.0000	0.3500	0.5813
6	0.3333	0.3474	0.5845	0.5676	0.4582
7	0.7552	0.8462	0.4742	0.3818	0.6143
8	0.7184	0.7674	0.4538	0.3559	0.5739
9	1.0000	1.0000	0.5134	1.0000	0.8783
Mean grey relational grade					0.6111

**Fig. 1:**
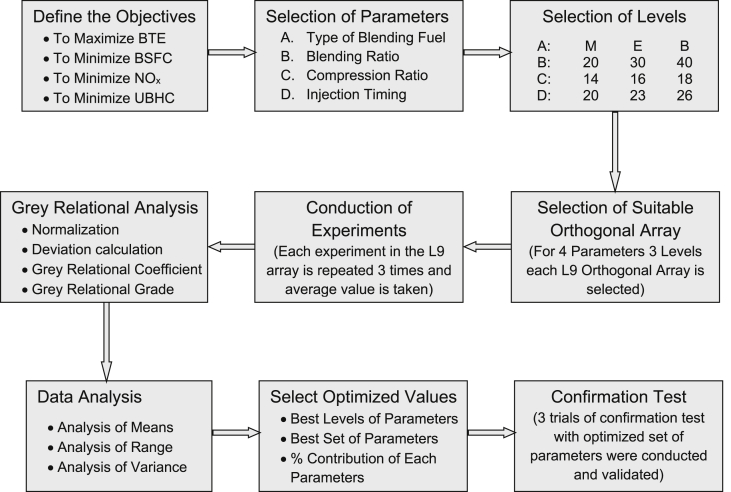
Flow chart representation of research methodology of the current work.

**Fig. 2:**
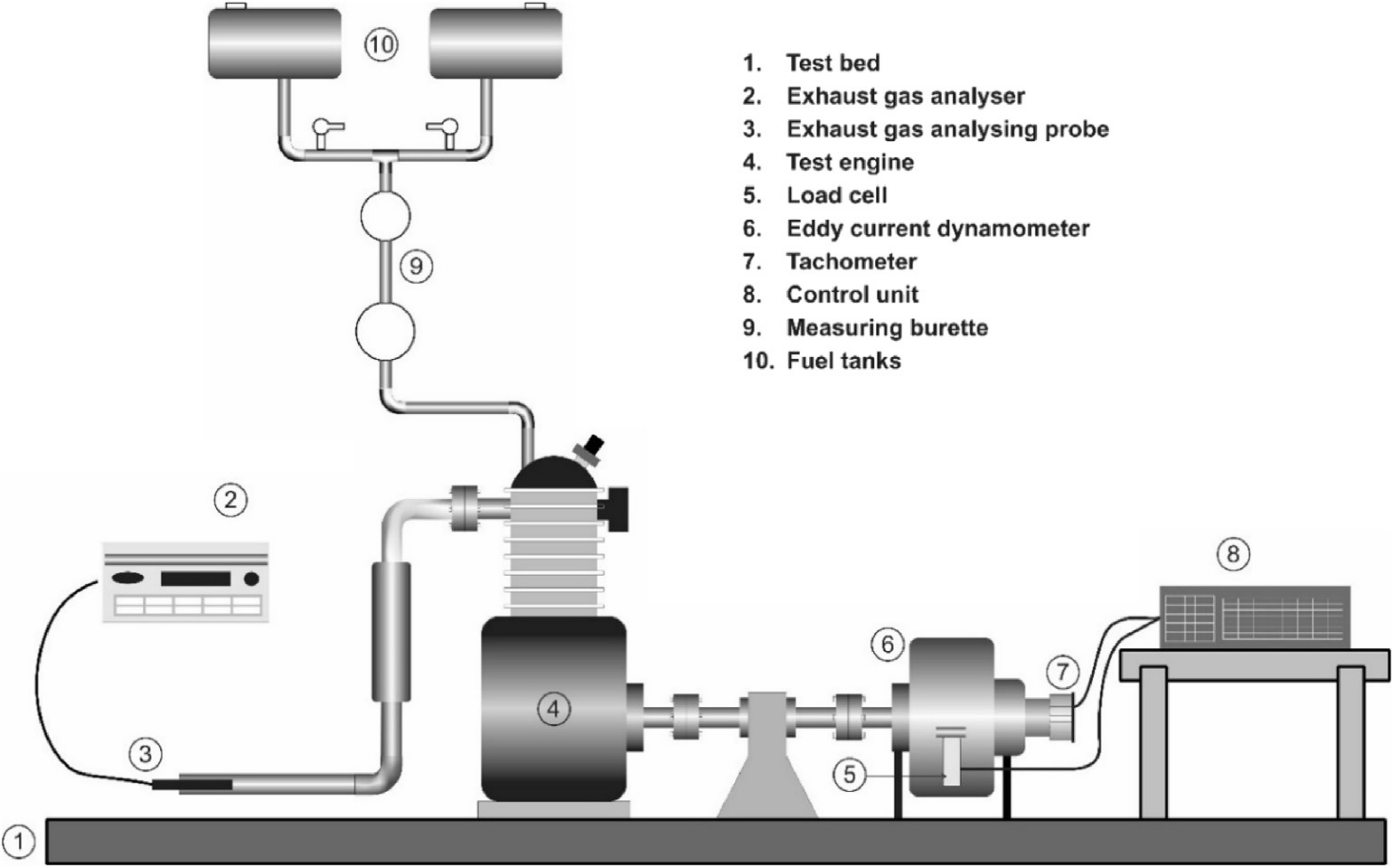
Schematic diagram of the experimental setup.

**Fig. 3:**
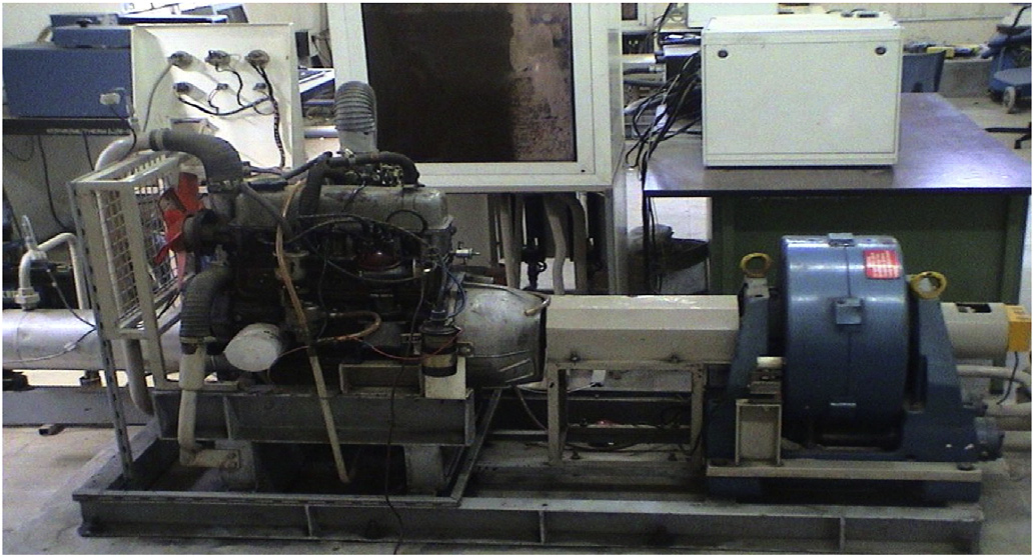
Photographic view of engine experimental setup.

**Fig. 4:**
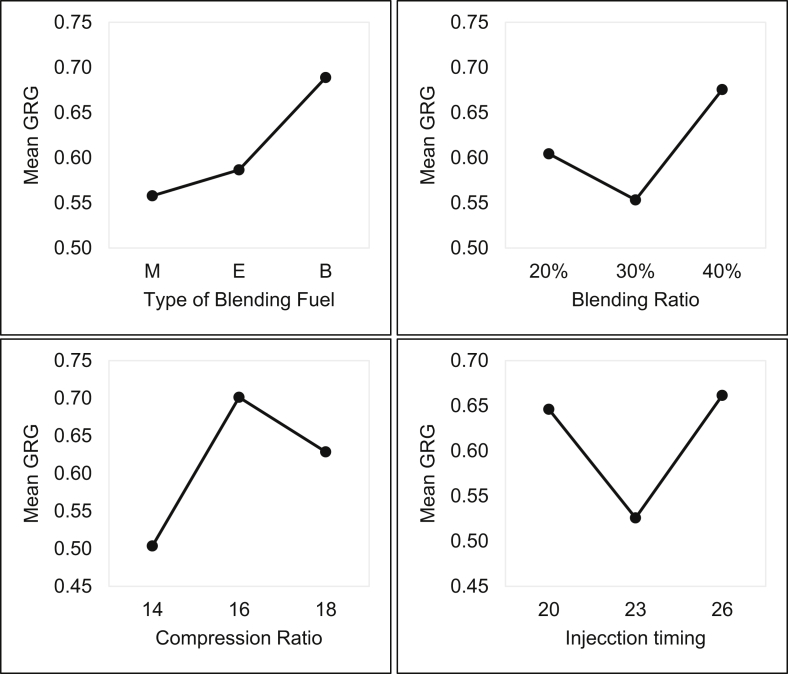
Mean grey relational grade graph for each parameters.

**Fig. 5:**
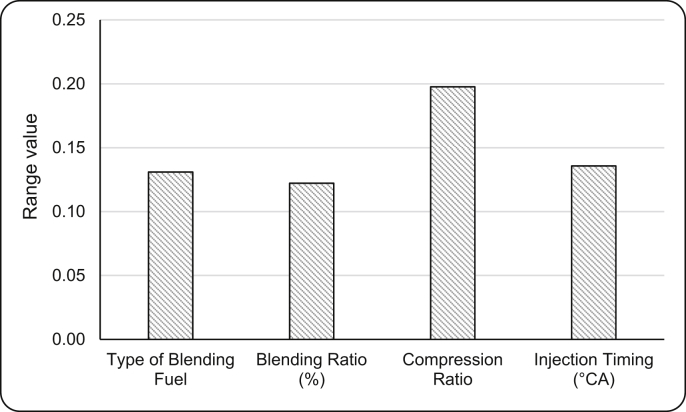
Range analysis of each parameter.

**Table 9:** Mean and range table for grey relational grade.

Levels	Parameters			
	Type of Blending Fuel	Blending Ratio (%)	Compression Ratio	Injection Timing (°CA)
1	0.5579	0.6044	0.5035	0.6461
2	0.5866	0.5533	0.7012^*^	0.5258
3	0.6889^*^	0.6756^*^	0.6286	0.6615^*^
Range (Rj)	0.1310	0.1223	0.1976	0.1357
Rank	3	4	1	2

The best level of each parameter is highlighted in bold letters.

**Table 10:** Response table for grey relational grade.

Parameters	SS_j_	% Contribution
Type of Blending Fuel	0.0284	19.72
Blending Ratio (%)	0.0226	15.70
Compression Ratio	0.0600	41.61
Injection Timing (°CA)	0.0331	22.97
***SS***_***T***_	0.1441	100

**Table 11:** Results of confirmation test.

		Normal operating conditions	Optimized operating conditions
Operating conditions	Blending fuel	Diesel	Butanol
	Blending percentage	40%	40%
	Compression ratio	16:1	16:1
	Injection timing	23 °CA BTDC	26 °CA BTDC
Results	BTE (%)	26.61	31.38
	BSFC (kg/kW-hr)	0.54	0.41
	NO_x_ (ppm)	763	656
	UBCH (ppm)	59	43

## 2 Experimental design, materials, and methods

The step by step methodology followed in this research work is shown in Fig. 1. In the first step the four objectives of the research work were fixed as higher brake thermal efficiency, lower brake specific fuel consumption, lower oxides of nitrogen emission and lower unburnt hydrocarbon emission. In the second step four process parameters (two engine parameters and two blending fuel parameters) were chosen. Next three levels of each parameters were fixed. The selected parameters and their levels were given in Table 1. Suitable orthogonal array was selected based on number of parameters and number of levels in the step 4 and given in Table 2. Designed experiments were carefully conducted and data were recorded [1]. To convert the multi-objective optimization problem in to single objective optimization problem grey relational analysis (GRA) was performed in step 6. GRA is a simple and accurate mathematical technique to find an appropriate solution for real time multivariate problems by computing grey relational grade. Once the grey relational grade was computed statistical analyses such as analysis of means, analysis of range and analysis of variance were performed in step 7. In the next step optimized set of parameters were selected [3]. In the final step a confirmation experiment was conducted and results were validated.

### 2.1 Chemicals and materials used

Raw pongamia pinnata oil was purchased from the local market, 85% pure laboratory grade potassium hydroxide in pellet form, concentrated sulphuric acid and methanol with 99% purity were used for preparation of biodiesel. 99% pure methanol, ethanol and butanol were purchased and used for engine experiments.

### 2.2 Preparation of biodiesel

The free fatty acid value of Pongamia pinnata oil was found to be 8.78%. To reduce the FFA value, first acid catalytic esterification was done with 6:1 M ration of methanol to oil and 1% (w/w) sulphuric acid. The FFA value was reasonably reduced to 1.35% and then transesterification was processed with 6:1 M ration of methanol to oil, 1% (w/w) KOH, 90 minutes reaction time, 60 °C process temperature, and stirring speed of 500 rpm. Then the products were settled in settling flask for 24 hours and pure biodiesel Pongamia Pinnata Methyl Ester (PPME) was separated from crude glycerol [4]. Physiochemical properties of prepared biodiesel, diesel, methanol, ethanol and butanol were tested using respective ASTM standard procedure for each property and listed in Table 3.

### 2.3 Experimental setup and procedure

The experimental setup consists of a Kirloskar single cylinder four stroke direct injection variable compression ratio engine coupled with an eddy current dynamometer. AVL 444 model 5 gas analyzer for measurement of various polutants from engine ehaust was also equipped with experimental setup. The detailed specification of the test engine is given in Table 4. The schematic and photographic views of test setup are shown in Fig. 2, Fig. 3 respectively. The list of instruments used, range of measurement, accuracy, measurement technique and percentage uncertainties are given in Table 5. A set of experiments given in L9 orthogonal array were conducted at rated full load, at the rated constant speed of 1500 rpm. Three repetitions of each experiment was conducted and the mean value of the trials has been taken into consideration. Once the engine has attained the rated speed during each experiment, time to consume 20 cm^3^ of fuel and emissions such as UBHC and NO_x_ were recorded [5], [6].

### 2.4 Grey relational analysis

Grey relational analysis is one of the reliable statistical method to convert multi-objective optimization problem in to single objective optimization problem. In GRA the data collected from experiments are known as original sequence. The original sequence collected from experiments for different objective performance characteristics are in different dimensions. They should be normalized between 0 and 1. The normalized value of the original sequence is called as comparability sequence. Subsequently, the deviation sequence is calculated from the comparability sequence. For calculating deviation sequence, reference sequence should first set as highest normalized value. Then the grey relational coefficient is calculated. In grey relational coefficient the value of distinguishing coefficient should be set any value between 0 and 1. The distinguishing coefficient can be adjusted by the decision maker exercising judgment and in this study it is set as 0.5. Finally the grey relational grade is computed by averaging the grey relational coefficients of all individual responses. Important relations used in grey relational analysis are given below [7].

The normalization can be done by the following relations,

For higher-the-better

(1) $$x_{i}^{*}\left(z\right)=\frac{x_{i}^{o}\left(z\right)-\min\;x_{i}^{o}\left(z\right)}{\max\;x_{i}^{o}\left(z\right)-\min\;x_{i}^{o}\left(z\right)}$$

For lower-the-better

(2) $$x_{i}^{*}\left(z\right)=\frac{\max\;x_{i}^{o}\left(z\right)-x_{i}^{o}\left(z\right)}{\max\;x_{i}^{o}\left(z\right)-\min\;x_{i}^{o}\left(z\right)}\;$$

The deviation sequence is computed using the following relation,

(3) $$\Delta_{oi}\left(z\right)=\left\|x_{o}^{*}\left(z\right)-x_{i}^{*}\left(z\right)\right\|$$

The grey relational coefficient is calculated using the following relation

(4) $$\xi_{i}\left(z\right)=\frac{\text{Δ}min+\zeta \text{Δ}max}{{\text{Δ}}_{oj}\left(z\right)+\zeta \text{Δ}max}\;$$

(5) $$\text{Δ}min=\underset{\forall i\in i}{\min\;}\underset{\forall k}{\min}\left\|x_{o}^{*}\left(z\right)-x_{i}^{*}\left(z\right)\right\|\;$$

(6) $$\text{Δ}max=\underset{\forall i\in i}{\max\;}\underset{\forall k}{\max}\left\|x_{o}^{*}\left(z\right)-x_{i}^{*}\left(z\right)\right\|$$

The grey relational grade is calculated using the following relation

(7) $$Y_{i}=\frac{1}{N}\sum_{z=1}^{N}\xi_{i}\left(z\right)$$

where.

z=1toN
i=1ton
N=Numberofperformacnecharacteristics
n=Numberofexperiments
$x_{i}^{o}\left(z\right)=Original\;sequence$
$x_{i}^{*}\left(z\right)=Normalized\;or\;comparability\;sequence$
$x_{o}^{*}\left(z\right)=Reference\;sequence$
$min\;x_{i}^{o}\left(z\right)=Smallest\;value\;of\;x_{i}^{o}\left(z\right)$
$max\;x_{i}^{o}\left(z\right)=Largest\;value\;of\;x_{i}^{o}\left(z\right)$
ζ=Distinguishingcoefficient(0≤ζ≤1)

### 2.5 Data analysis for optimization

Once the grey relational grade has been computed some statistical analysis such as analysis of means, analysis of range and analysis of variance for identifying best set of parameters for combined optimization were carried out. Analysis of means (ANOM) is one of the important analysis to find out the optimal level of each parameters and optimal set of parameters. Mean values of each parameter at different levels can be represented as ${\bar{Y}}_{jk}$. In this research work for *L*_*9*_ orthogonal array of four parameters and three levels each, *j* represents parameters (*j = A,B,C,D*) and *k* represents levels (*k = 1,2,3*). Mean values of each parameter at different levels can be calculated using the following relations.

(8) $$\begin{array}{l} \begin{array}{ccc} Y_{A1}=Y_{1}+Y_{2}+Y_{3}; & Y_{A2}=Y_{4}+Y_{5}+Y_{6}; & Y_{A3}=Y_{7}+Y_{8}+Y_{9}\\ Y_{B1}=Y_{1}+Y_{4}+Y_{7}; & Y_{B2}=Y_{2}+Y_{5}+Y_{8}; & Y_{B3}=Y_{3}+Y_{6}+Y_{9}\\ Y_{C1}=Y_{1}+Y_{6}+Y_{8}; & Y_{C2}=Y_{2}+Y_{4}+Y_{9}; & Y_{C3}=Y_{3}+Y_{5}+Y_{7} \end{array}\\ Y_{D1}=Y_{1}+Y_{5}+Y_{9};\begin{array}{ccc} & Y_{D2}=Y_{2}+Y_{6}+Y_{7}; & Y_{D3}=Y_{3}+Y_{4}+Y_{8} \end{array}\\ {\bar{Y}}_{jk}=\frac{Y_{jk}}{3} \end{array}$$

where $Y_{1},Y_{2,}\ldots Y_{9}$ are response dependent variable (% yield of biodiesel) of experiment numbers *1, 2 … 9* respectively. The maximum mean value of particular parameter at particular level indicates the best level of that parameter and the combination of highest mean values of each parameter will give the optimal parameter combination. Range analysis is used to identify which parameter will highly influence the dependent variable and assign rank accordingly. The range values of different parameters can be calculated using the following relation

(9) $$R_{j}=\;\max\;{\bar{Y}}_{jk}-\;\min\;{\bar{Y}}_{jk}$$

The larger range value is the indication of higher influence of that parameter on dependent variable, hence assigned rank 1 and second larger will be assigned rank 2 and so on. Range analysis will be helpful in ranking the individual parameters based on their influence on response variable, but it will not be helpful in quantifying the percentage contribution of each parameter on the dependent variable. This limitation will be overcome by performing Analysis of Variance (ANOVA) of the experimental data.

(10) $$SS_{j}=\left(\frac{1}{3}\sum_{k=1}^{3}Y_{jk}^{2}\right)-\frac{1}{n}{\left(\sum_{i=1}^{n}Y_{i}\right)}^{2}$$

(11) $$SS_{T}=\left(\sum_{i=1}^{n}{Y_{i}}^{2}\right)-\frac{1}{n}{\left(\sum_{i=1}^{n}Y_{i}\right)}^{2}$$

(12) $$\%\;contribution\;of\;j^{th}\;parameter=\;\frac{SS_{j}}{SS_{T}}\times 100$$

Where

$SS_{T}-\;\text{Total}\;\text{sum}\;\text{of}\;\text{squares}$
$SS_{j\;}-\;\text{Sum}\;\text{of}\;\text{squares}\;\text{of}\;j^{th}\;\text{parameter}\;$
i=1,2,3…n
k=1,2,3
j=A,B,C,D
n−NumberofExperiments

The experimental conditions and the experimental results or response sequence or original sequence of four objective functions are given in Table 6. The normalized sequence and their corresponding deviation sequence are given in Table 7. The computed grey relational coefficients and grey relational grade are given in Table 8. The mean analysis was carried out for grey relational grade using equation (8). The range values were computed and rank was assigned correspondingly using equation (9). The mean, range and rank values are given in Table 9. The mean grey relational grade graph and range graph are represented in Fig. 4, Fig. 5 respectively. The percentage contributions for each parameter was computed and given in Table 10. The results of confirmation test was compared with results of normal operating condition of engine with 60% PPME and 40% diesel bend (given in Table 11) and found that 17.93% increase in BTE, 24.07% decrease in BSFC, 14.02% decrease in NO_x_ and 27.12% decrease in UBHC were recorded.
